# Effects of combined d-fagomine and omega-3 PUFAs on gut microbiota subpopulations and diabetes risk factors in rats fed a high-fat diet

**DOI:** 10.1038/s41598-019-52678-5

**Published:** 2019-11-12

**Authors:** Mercè Hereu, Sara Ramos-Romero, Cristina Busquets, Lidia Atienza, Susana Amézqueta, Bernat Miralles-Pérez, Maria Rosa Nogués, Lucía Méndez, Isabel Medina, Josep Lluís Torres

**Affiliations:** 1 Institute of Advanced Chemistry of Catalonia (IQAC-CSIC), Barcelona, Spain; 2 Department of Cell Biology, Physiology & Immunology, Faculty of Biology, University of Barcelona, Barcelona, Spain; 3 Department of Pathology Puerta del Mar University Hospital, Cádiz, Spain; 4 Departament d’Enginyeria Química i Química Analítica and Institut de Biomedicina (IBUB), Universitat de Barcelona, Barcelona, Spain; 5 Universitat Rovira i Virgili, Unitat de Farmacologia, Facultat de Medicina i Ciències de la Salut, Reus, Spain; 6 Instituto de Investigaciones Marinas (IIM-CSIC), Vigo, Spain

**Keywords:** Pre-diabetes, Nutrition

## Abstract

Food contains bioactive compounds that may prevent changes in gut microbiota associated with Westernized diets. The aim of this study is to explore the possible additive effects of d-fagomine and ω-3 PUFAs (EPA/DHA 1:1) on gut microbiota and related risk factors during early stages in the development of fat-induced pre-diabetes. Male Sprague Dawley (SD) rats were fed a standard diet, or a high-fat (HF) diet supplemented with d-fagomine, EPA/DHA 1:1, a combination of both, or neither, for 24 weeks. The variables measured were fasting glucose and glucose tolerance, plasma insulin, liver inflammation, fecal/cecal gut bacterial subgroups and short-chain fatty acids (SCFAs). The animals supplemented with d-fagomine alone and in combination with ω-3 PUFAs accumulated less fat than those in the non-supplemented HF group and those given only ω-3 PUFAs. The combined supplements attenuated the high-fat-induced incipient insulin resistance (IR), and liver inflammation, while increasing the cecal content, the Bacteroidetes:Firmicutes ratio and the populations of Bifidobacteriales. The functional effects of the combination of d-fagomine and EPA/DHA 1:1 against gut dysbiosis and the very early metabolic alterations induced by a high-fat diet are mainly those of d-fagomine complemented by the anti-inflammatory action of ω-3 PUFAs.

## Introduction

There is mounting evidence that diet affects homeostasis by modifying the gut microbial populations in both rodents and humans^1–3^. Early insulin resistance (IR) and impaired glucose tolerance (IGT), which appear in the first stage of diet-induced type 2 diabetes^4^, might be triggered by intestinal barrier alterations induced by unbalanced microbiota (dysbiosis)^5^. Lipopolysaccharides (LPS: a component of the cell wall of Gram-negative bacteria), short-chain fatty acids (SCFAs: end products of the fermentation of dietary fiber by bacteria) and bile acids are possible mediators linking gut bacteria to IR and adipose tissue function^5^. Dietary changes can rapidly modify gut microbial composition^1,6^. Fat- or carbohydrate-restricted diets increase the populations of the order Bacteroidetes while reducing body weight^7^. At the genus level, a high intake of fat and protein is positively associated with *Bacteroides*; whereas a high fiber intake is related to increased levels of *Prevotella*^8,9^. Food components such as prebiotic fiber may prevent changes in gut microbiota associated with obesity and metabolic disorders^2^.

The iminosugar d-fagomine (1,2-dideoxynojirimycin) is a stable cyclic carbohydrate analog with a nitrogen atom in place of the oxygen atom within the cycle that was first isolated from buckwheat seeds (*Fagopyrum esculentum*)^10^. d-Fagomine reduces post-prandial blood glucose concentration in sucrose/starch loading tests in healthy rats through the inhibition of intestinal disaccharidases^11^ and it attenuates high-fat-induced low-grade inflammation and IGT in rats^12,13^. d-Fagomine also promotes gut microbial diversity by increasing the relative populations of Bacteroidetes in healthy rats, while also mitigating the age-related reduction in the populations of the putatively beneficial bacteria Lactobacillus and Bifidobacterium^14^ and stabilizing the populations of *Prevotella* in the intestinal tract of healthy rats (Hereu, *Nutrients*, accepted).

Eicosapentaenoic acid (EPA, 20:5, n-3) and docosahexaenoic acid (DHA, 22:6, n-3) are the major ω-3 PUFAs in fish oil. EPA and DHA reduce elevated plasma cholesterol and triglycerides, oxidative stress (OS), and high blood pressure, which are risk factors for cardiovascular diseases^15^ and other pathologies that involve inflammation^16^. The possible contribution of changes in gut microbiota to the anti-inflammatory activity of ω-3 PUFAs is poorly documented. It has been shown that EPA and DHA increase the populations of putatively beneficial gut Lactobacillus and Bifidobacteria in mice fed a high-fat diet^17,18^. When combined with proanthocyanidins or d-fagomine, EPA/DHA 1:1 helps to stabilize the populations of Bifidobacteriales and Lactobacilliales in healthy rats^19^.

The aim of this study is to explore the possible complementary or additive effects of d-fagomine and ω-3 PUFAs (EPA/DHA 1:1) on gut microbiota and risk factors for diabetes during early stages in the development of fat-induced pre-diabetes in rats.

## Methods

### Animals, experimental design and sample collection

Male Sprague Dawley (SD) rats from Envigo (Indianapolis, IN, USA), aged 8–9 weeks were used. As we wanted to detect early effects of the supplementations, SD rats were preferred over other strains, such as Wistar rats, because they take longer to develop IGT^20^. The rats (n = 45) were housed (n = 3 per cage) under controlled conditions of humidity (60%), and temperature (22 ± 2 °C) with a 12 h light-12 h dark cycle. They were randomly divided into 5 groups (n = 9 per group): the control group (STD), fed a standard diet (2014 Teklad Global 14% Protein Diet from Envigo); the high-fat group (HF), fed only a high-fat diet (TD.08811 45% kcal fat diet from Envigo) with no supplementation; a group fed the high-fat diet supplemented with d-fagomine (Envigo custom designed, manufactured by Mucedola srl; Settimo Milanese, Italy) (HF + FG group); a group fed the high-fat diet supplemented with ω-3 PUFAs (HF + ω-3 group); and a group fed the high-fat diet supplemented with both d-fagomine and ω-3 PUFAs (HF + FG + ω-3 group). d-Fagomine (>98%) manufactured by Bioglane SLNE (Barcelona, Spain) was generously provided by Taihua Shouyue (HK) International Co. Ltd. (Hong Kong, China). It was included in the feed at a proportion of 0.96 g/kg feed, as in previous studies^11,13^. ω-3 PUFAs (EPA/DHA 1:1) were obtained by mixing the appropriate quantities of the commercial fish oils AFAMPES 121 EPA (AFAMSA, Vigo, Spain) and EnerZona Omega 3 RX (Milan, Italy). ω-3 PUFAs were administered by oral gavage using a gastric probe once a week at a dose of 0.8 mL oil per kg body weight. The dose and EPA/DHA proportions used were previously determined^21^. To compensate for the stress of probing and the excess of calories from fish oil in the HF + ω-3 and HF + FG + ω-3 groups, the animals in the groups STD, HF and HF + FG were administered soybean oil at the same dose at the same time. The major fatty acids in soybean oil triglycerides are polyunsaturated alpha-linolenic acid (7%) and linoleic acid (LA, 51%), monounsaturated oleic acid (23%) and the saturated stearic and palmitic acids (4% and 11%, respectively)^22^. All the groups were fed *ad libitum* with free access to water. The composition of the diets is provided as Supplementary Material, Table 1.

Feed consumption was monitored daily and body weight was measured weekly throughout the experiment. Energy intake was calculated as estimates of metabolizable energy based on the Atwater factors, assigning 4 kcal/g protein, 9 kcal/g fat, and 4 kcal/g available carbohydrate.

After overnight fasting, blood samples were collected from the saphenous vein at weeks 9, 14 and 23 of the experiment. Plasma was separated by centrifugation and stored at −80 °C until analysis. At week 21, urine was collected by placing the rats in metabolic cages; while after week 23, fecal samples were collected by abdominal massage.

At the end of the experiment (week 24), the rats were fasted overnight and anesthetized intraperitoneally with ketamine and xylazine (80 and 10 mg per kg body weight, respectively) and then killed by exsanguination. Perigonadal fat, liver and cecum were removed, weighed and stored at −80 °C. One portion of the liver was fixed in 4% formalin for histological analysis.

All the procedures strictly adhered to the European Union guidelines for the care and management of laboratory animals, and were licensed by the regional Catalan authorities (reference no. DAAM7921), as approved by the Spanish CSIC Subcommittee of Bioethical Issues.

### Glycemic status

Fasting blood glucose and plasma insulin levels were measured after weeks 9, 14 and 23 in fasted animals. Blood glucose concentration was measured by the enzyme electrode method, using an Ascensia ELITE XL blood glucose meter (Bayer Consumer Care; Basel, Switzerland). Plasma insulin levels were measured using the rat/mouse insulin ELISA kit from Millipore Corporation (Billerica, MA, USA). The HOMA (Homeostatic Assessment Model) index was calculated as fasting insulin (µU/mL) × fasting glucose (mmol/L)/22.5^23^. Insulin units (IU) were calculated using the conversion 1 IU = 0.0347 mg insulin.

The oral glucose tolerance test (OGTT) was performed at week 20 on fasted animals. A solution of glucose (1 g/kg body weight) was administered by oral gavage, and blood glucose concentration was measured 15, 30, 45, 60, 90 and 120 min after glucose intake by the enzyme electrode method.

Plasma leptin levels were measured using MILLIPLEX xMAP multiplex technology on a Luminex xMAP instrument (Millipore, Austin, TX, USA) at week 23. MILLIPLEX Analyst 5.1 (VigeneTech; Carlisle, PA, USA) software was used for data analysis.

### Isoprostanes in urine

F_2t_-isoprostanes (F_2t_-IsoPs) were determined in urine samples by LC/ESI-MS/MS following a previously reported procedure^24^ with some modifications. Urine samples (500 µL) were acidified, β-glucuronidase (90 U/mL) (Sigma; Saint Louis, MO, USA) was added, and the mixtures were incubated for 2 h at 37 °C. After the addition of the internal standard [^2^H_4_]15-F_2t_-IsoP (Cayman; Ann Arbor, MO, USA) (100 µL, 10 µg/L), F_2t_-IsoPs were purified by SPE in a C18 Sep-Pak cartridge (Waters, Mildford, MA, USA). F_2t_-IsoPs were analyzed using an Agilent 1260 chromatograph fitted with a Mediterranea Sea 18 column (10 cm × 2.1 mm id., 2.2 µm particle size) (Teknokroma; Barcelona, Spain) coupled to a 4000 QTRAP mass spectrometer (Applied Biosystems; Foster City, CA, USA). The instrument was operated in the negative ion mode with a Turbo V source to obtain MS/MS data. Separation was achieved with a binary system consisting of 0.1% aqueous formic acid [A] and formic acid in acetonitrile [B], at 40 °C, with an increasing linear gradient (v/v) of [B]: 0 min, 10% B; 7 min, 50% B; 7.1 min, 100% B; 8 min, 100% B; 8.1 min, 10% B; and 10 min, 10% B, at a flow rate of 700 µL/min. F_2t_-IsoPs were detected by MS/MS multiple reaction monitoring. Calibration curves were prepared using seven matrix-matched standards covering the working concentration range. The LOQ was 0.4 µg/L for 15-F_2t_-IsoP and 2 µg/L for 5-F_2t_-IsoP. The results were expressed as nanograms per milligram of creatinine, to correct for urine dilution. Creatinine levels in urine were determined via a colorimetric method using a commercial kit (Creatinine-J, Spinreact; Girona, Spain) by measuring absorbance at 492 nm.

### Subpopulations of gut microbiota

The relative populations of selected bacterial phyla, orders and genera were estimated in fecal and cecal DNA by quantitative real-time polymerase chain reaction (qRT-PCR). DNA was extracted from fecal and cecal samples using a QIAamp® DNA Stool Mini Kit from QIAGEN (Hilden, Germany). Its concentration was quantified using a Nanodrop 8000 Spectrophotometer (ThermoScientific; Waltham, MA, USA) and all DNA samples were diluted to 20 ng/µL. The qRT-PCR experiments were carried out using a LightCycler® 480 II (Roche, Basel, Switzerland) in triplicate. Each qRT-PCR well contained a total of 20 µL: DNA (2 µL) and a master mix (18 µL) made of 2X SYBR (10 µL), the corresponding forward and reverse primer (1 µL each), and water (6 µL) purified using a Milli-Q system (Millipore Corporation; Billerica, MA, USA). All the reactions were paralleled by analysis of a non-template control (water) and a positive control (Supplementary Material, Table 2) from DSMZ (Braunschweig, Germany). The qRT-PCR cycling conditions were: 10 s at 95 °C, then 45 cycles of 5 s at 95 °C, 30 s at the primer-specific annealing temperature (Supplementary Material, Table 2), and 30 s at 72 °C (extension). The specificity of the qRT-PCR reactions was assessed by melting curve analysis which consisted of heating to 95 °C and maintaining this temperature for 2 s, then cooling to 65 °C and maintaining this temperature for 15 s, and running a temperature gradient from 65 °C to 95 °C at a rate of 0.11 °C/s, with five fluorescence recordings per °C. The relative DNA abundances for each bacterial subgroup were calculated from the second derivative maximum of their respective amplification curves (C*p*, calculated in triplicate) by considering C*p* values to be proportional to the dual logarithm of the inverse of the specific DNA concentration, following the equation: [DNA_a_]/[DNA_b_] = 2^Cpb-Cpa^ ^25^. Amounts of total bacteria were normalized as 16S rRNA gene copies per mg of wet feces (copies/mg).

### Short-chain fatty acids

SCFAs were analyzed in feces and in cecal content by gas chromatography (GC) using a previously described method^26^ with some modifications. Briefly, the samples were freeze-dried and weighed (~50 mg dry matter) and a solution (1.5 mL) containing the internal standard 2-ethylbutiric acid (6.67 mg/L) and oxalic acid (2.97 g/L) in acetonitrile/water 3:7 was added. Then, SCFAs were extracted for 10 min using a rotating mixer. The suspension was centrifuged (5 min, 12,880 g) in a 5810R centrifuge (Eppendorf; Hamburg, Germany) and the supernatant passed through a 0.45 µm nylon filter. An aliquot of the supernatant (0.7 mL) was diluted to 1 mL with acetonitrile/water 3:7. SCFAs were analyzed using a Trace 2000 gas chromatograph coupled to a flame ionization detector (ThermoFinnigan; Waltham, MA, USA) equipped with an Innowax 30 m × 530 µm × 1 µm capillary column (Agilent; Santa Clara, CA, USA). Chrom-Card software was used for data processing. Helium was used as the carrier gas with a linear velocity of 5 mL/min. GC oven temperature was programmed as follows: 80 °C (hold 1 min) to 120 °C at 15 °C/min (hold 4 min) to 130 °C at 5 °C/min (hold 4 min) to 235 °C at 8 °C/min (hold 4 min). Flame ionization detection (FID) was performed at a base temperature of 240 °C. Calibration curves were prepared using seven matrix-matched standards covering the working concentration range. The precision (RSD <15%) and recovery (>70%) of the method were adequate and both inter- and intra-day reproducible.

### Liver histology

Fixed livers were dehydrated in alcohol and embedded in paraffin (Panreac Quimica SLU; Barcelona, Spain), then cut into 3 µm-thick slices, using a steel knife mounted in a microtome (HM 355S Rotary Microtome, Thermo Fisher Scientific; Waltham, MA, USA). Sections were stained with hematoxylin (hematoxylin solution modified in accordance with Gill III for microscopy, Merck KGaA; Darmstadt, Germany)/eosin (Pharmacy Service of Puerta del Mar Hospital; Cadiz, Spain) then viewed under a light microscope (NIKON Eclipse 80i, NIKON Corporation; Minato, Japan). Variables were graded following the method described by Taltavull *et al*.^27^ using observation of the entire field of the tissue preparations: steatosis, 0 (absence) or 1 (presence); and lobular inflammation, 0 (absence), 1 (1–2 foci), 2 (2–4 foci), or 3 (>4 foci).

### Statistical analysis

All data manipulation and statistical analysis was performed using GraphPad Prism 5 (GraphPad Software; San Diego, CA, USA). The results are expressed as means with their standard errors (SEM). The normal distributions and heterogeneity of the data were evaluated, and statistical significance was determined by one-way ANOVA and the Tukey multiple-comparisons test or by two-way ANOVA. Differences were considered significant when *P* ≤ 0.05 and were considered to indicate a tendency when 0.05 < *P* ≤ 0.1.

## Results

### Feed and energy intakes, body weight, perigonadal fat, plasma leptin and urine F_2t_-isoprostanes

All the rats fed the high-fat diet consumed less feed but more energy than those in the STD group (Table 1), whether they were supplemented with d-fagomine and/or ω-3 PUFAs or not.

**Table 1:** Feed and energy intakes, and final body weight gain of SD rats fed a high-fat diet supplemented, or not, with d-fagomine, ω-3 PUFAs or a combination of both, for 24 weeks.

	STD		HF		HF + FG		HF + ω-3		HF + FG + ω-3	
	Mean	SEM	Mean	SEM	Mean	SEM	Mean	SEM	Mean	SEM
Feed intake (g/day/100 g body weight)	4.6	0.5	3.3***	0.1	3.0***	0.1	3.2***	0.1	3.4***	0.1
Energy intake^£^ (kcal/day/100 g body weight)	13.3	1.4	15.3***	0.5	15.0***	0.1	15.7***	0.5	16.1***	0.1
Body weight gain (%)	67.3	4.1	78.9^†^	4.6	74.2	2.2	76.4	5.7	71.5^φ^	3.3

^£^Energy intake is estimated as metabolizable energy based on Atwater factors, which assign 4 kcal/g protein, 9 kcal/g fat, and 4 kcal/g available carbohydrates.

Data are presented as means with their standard errors; n = 9 per group. Comparisons were conducted using one-way ANOVA and Tukey’s multiple comparisons test. ^***^P < 0.001 *vs* STD (^†^P = 0.07 *vs* STD, ^φ^P = 0.08 *vs* HF).

At the beginning of the study, the mean body weight was 323.9 ± 6.3 g (Supplementary Material, Fig. 1). At the end of the study (after 24 weeks) the rats in the HF group showed a tendency (P = 0.07) to gain more weight than the STD group (Table 1). There were no significant differences between the supplemented groups and either the STD or the HF group. The body weight curve corresponding to the group supplemented with both d-fagomine and ω-3 PUFAs was similar to that of the STD group without reaching statistically significant differences with that of the HF group (Supplementary Material, Fig. 1B).

**Figure 1:**
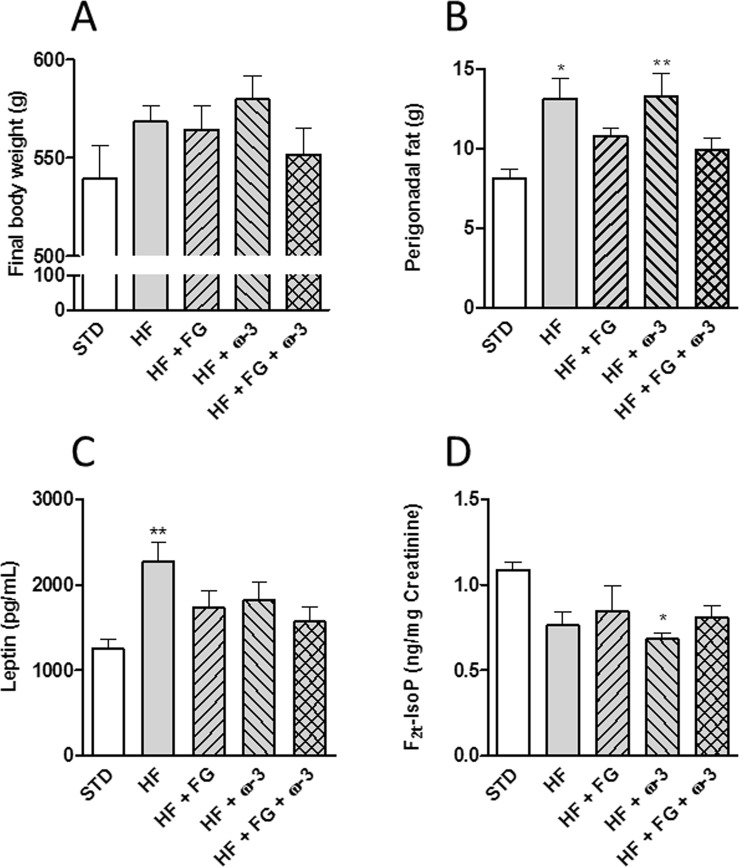
Final body weight (**A**) perigonadal fat (**B**) plasma leptin (**C**) and urine F_2t_-IsoPs (**D**) of SD rats fed a standard (STD), high-fat (HF), high-fat supplemented with d-fagomine (HF + FG), high-fat supplemented with EPA/DHA (1:1) (HF + ω-3) or high-fat supplemented with both d-fagomine and EPA/DHA (1:1) (HF + FG + ω-3) diet for 24 weeks (**A**–**C**) or 21 weeks. (**D**) Data are presented as means with their standard errors. Comparisons were performed using one-way ANOVA followed by Tukey’s post-hoc test. *P < 0.05, **P < 0.01 *vs* STD.

The HF group and the rats supplemented only with ω-3 PUFAs showed significantly higher (P < 0.05) perigonadal fat deposition than the STD group; while the groups supplemented with d-fagomine presented a level of perigonadal fat similar to the STD group (Fig. 1B).

Plasma leptin concentration (Fig. 1C) was higher (P < 0.01) in the HF group than in the STD group; all the supplementations resulted in leptin levels statistically similar to those of the STD group.

The animals fed the high-fat diet supplemented with ω-3 PUFAs had significantly (P < 0.05) lower concentrations of total urine F_2t_-IsoPs (5-F_2t_-IsoP plus 15-F_2t_-IsoP: markers of systemic OS) after 21 weeks, than rats in the STD group (Fig. 1D). The levels of F_2t_-IsoPs were similar among the supplemented groups.

### Glycemic status

**Figure 2:**
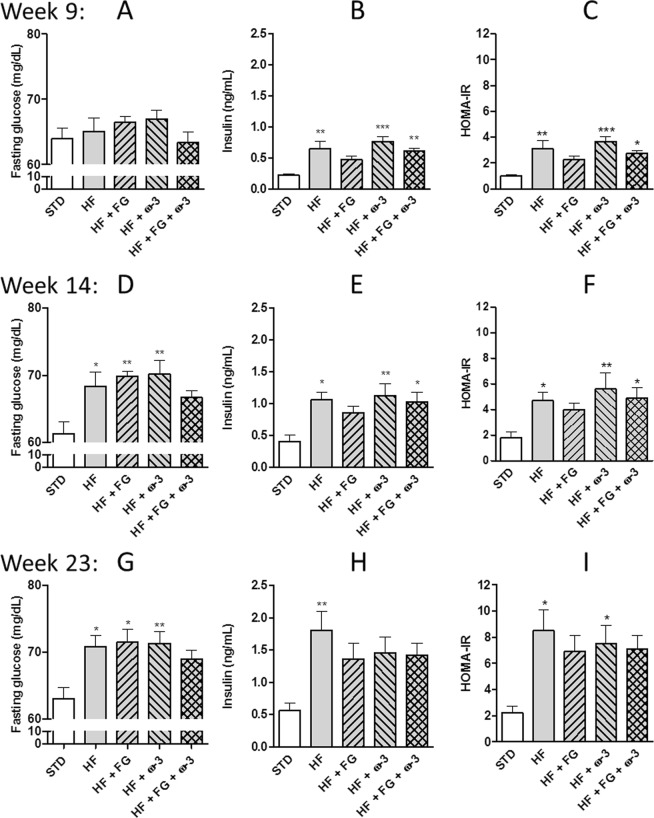
Plasma levels of fasting glucose (**A**,**D**,**G**) insulin (**B**,**E**,**H**) and calculated HOMA-IR (**C**,**F**,**I**) in SD rats fed a standard (STD), high-fat (HF), high-fat supplemented with d-fagomine (HF + FG), high-fat supplemented with EPA/DHA (1:1) (HF + ω-3) or high-fat supplemented with both d-fagomine and EPA/DHA (1:1) (HF + FG + ω-3) diet after 9 (**A**–**C**), 14 (**D**,**E**,**G**) and 23 weeks. (**H**–**J**) Data are presented as means with their standard errors. Comparisons were performed using one-way ANOVA followed by Tukey’s post-hoc test. *P < 0.05, **P < 0.01, ***P < 0.001 *vs* STD.

Plasma fasting glucose and insulin were measured at weeks 9, 14 and 23 (Fig. 2) of the study. Fasting glucose levels were below 80 mg/dL in all the groups at all times. Fasting glucose levels in the HF groups were higher (P < 0.05) than those in the STD group after 14 weeks of intervention (Fig. 2D,G), except in the group supplemented with both d-fagomine and ω-3 PUFAs; while plasma insulin was significantly elevated in the HF group compared to the STD group already at week 9 (Fig. 2B) and remained so up to the end of the study (Fig. 2H). The group supplemented only with d-fagomine presented insulin levels similar to the STD group throughout the experiment (Fig. 2B,E,H). The rats supplemented with ω-3 PUFAs (HF + ω-3 and HF + FG + ω-3 groups) showed plasma insulin levels similar to those in the STD group only at the end of the study (Fig. 2H).

HOMA-IR is an indicator of IR that accounts for the levels of fasting plasma glucose and insulin levels. HOMA-IR increased in all the HF groups (P < 0.05 *vs* STD group) except in the group supplemented with d-fagomine, as early as week 9 and for the entire experiment (Fig. 2C). At the end of the study (week 23), there were no significant differences in HOMA-IR between the groups supplemented with d-fagomine (HF + FG and HF + FG + ω-3) and the other groups (Fig. 2I).

The OGTT was performed at week 20, near the end of the experiment (Supplementary Material, Fig. 2). Although there were no significant differences in the area under the curve between groups, the response curves corresponding to the animals given the combination of d-fagomine and ω-3 PUFAs was superimposable to that generated with the control animals (Supplementary Material, Fig. 2C).

### Subpopulations of gut microbiota

**Figure 3:**
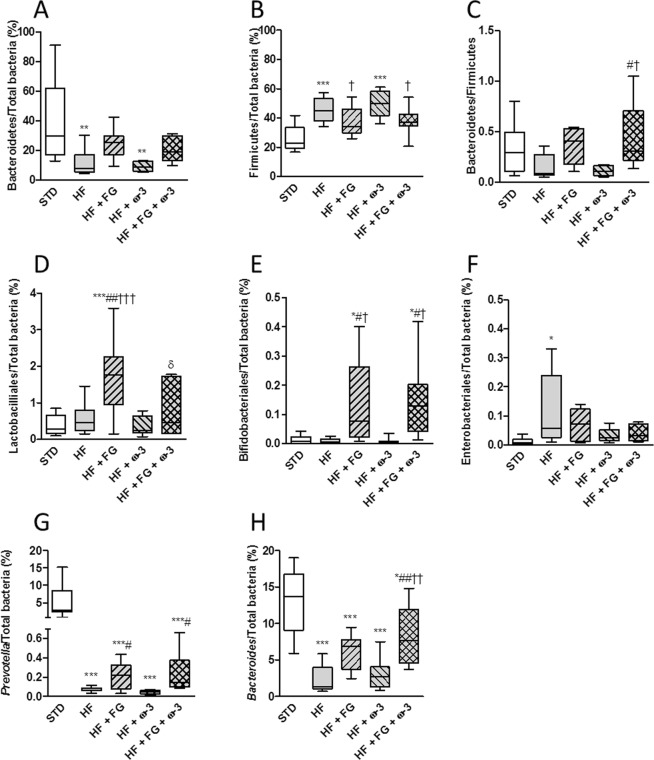
Relative gut microbial subpopulations in fecal samples from SD rats fed a standard (STD), high-fat (HF), high-fat supplemented with d-fagomine (HF + FG), high-fat supplemented with EPA/DHA (1:1) (HF + ω-3) or high-fat supplemented with both d-fagomine and EPA/DHA (1:1) (HF + FG + ω-3) diet after 23 weeks. Bacteroidetes (**A**) Firmicutes (**B**) Bacteroidetes:Firmicutes ratio (**C**) Lactobacilliales (**D**) Bifidobacteriales (**E**) Enterobacteriales (**F**) *Prevotella* (**G**) and *Bacteroides*. (**H**) Data are presented as means with their standard error. Comparisons were made using one-way ANOVA followed by Tukey’s post-hoc test. *P < 0.05, **P < 0.01, ***P < 0.001 *vs* STD; ^#^P < 0.05, ^##^P < 0.01, ^###^P < 0.001 *vs* HF; ^δ^P < 0.05 *vs* HF + FG; and ^†^P < 0.05, ^††^P < 0.01, ^†††^P < 0.001, *vs* HF + ω-3.

**Figure 4:**
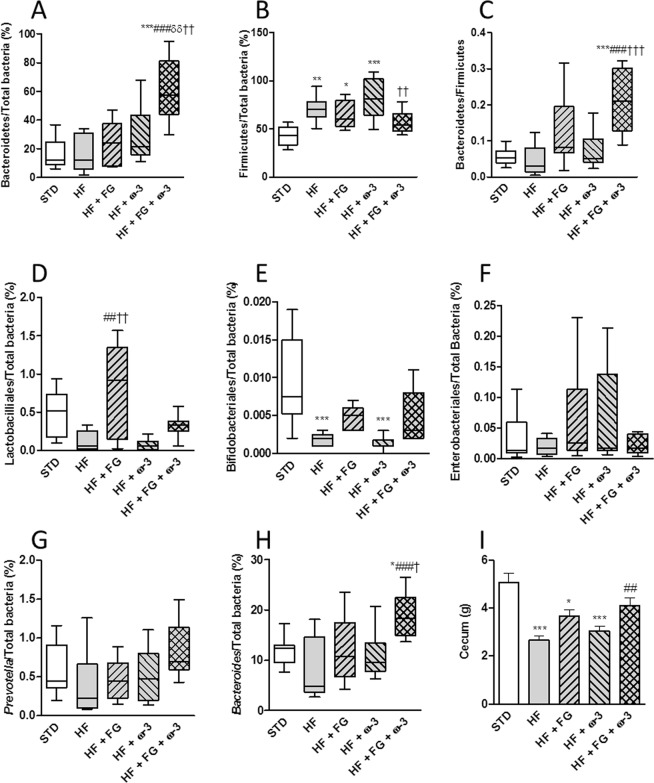
Relative gut microbial subpopulations in cecal samples of SD rats fed a standard (STD), high-fat (HF), high-fat supplemented with d-fagomine (HF + FG), high-fat supplemented with EPA/DHA (1:1) (HF + ω-3) or high-fat supplemented with both d-fagomine and EPA/DHA (1:1) (HF + FG + ω-3) diet after 24 weeks. Bacteroidetes (**A**) Firmicutes (**B**) Bacteroidetes:Firmicutes ratio (**C**) Lactobacilliales (**D**) Bifidobacteriales (**E**) Enterobacteriales (**F**) *Prevotella* (**G**) and *Bacteroides* (**H**) Cecum weight (**I**). Data are presented as means with their standard error. Comparisons were made using one-way ANOVA followed by Tukey’s post-hoc test. *P < 0.05, **P < 0.01, ***P < 0.001 *vs* STD; ^##^P < 0.01, ^###^P < 0.001 *vs* HF; ^δδ^P < 0.01 *vs* HF + FG; and ^†^P < 0.05, ^††^P < 0.01, ^†††^P < 0.001 *vs* HF + ω-3.

The proportions of the major bacterial phyla (Bacteroidetes and Firmicutes), selected orders (Lactobacilliales, Bifidobacteriales and Enterobacteriales) and genera (*Prevotella* and *Bacteroides*) were estimated in fecal and cecal DNA (Figs 3 and 4, respectively) at the end of the study (week 23–24).

In fecal samples, the proportion of Bacteroidetes (Fig. 3A) significantly (P < 0.01) decreased and the proportion of Firmicutes (Fig. 3B) significantly (P < 0.001) increased in rats from groups HF and HF + ω-3, with respect to the STD group. The proportion of Bacteroidetes and Firmicutes in animals supplemented with d-fagomine (the HF + FG and HF + FG + ω-3 groups) was similar to that in animals in the STD group. The percentage of Lactobacilliales (Fig. 3D) was significantly (P < 0.05) higher in the HF + FG group than in all other groups; while the population of Bifidobacteriales (Fig. 3E) was significantly (P < 0.05) higher in both groups supplemented with d-fagomine (HF + FG and HF + FG + ω-3) compared to the other groups. Only the HF group presented a proportion of Enterobacteriales significantly (P < 0.05) higher than that in the STD group (Fig. 3F). Significant differences between groups in the relative populations of the genera *Prevotella* and *Bacteroides* were detected. The proportions of *Prevotella* (Fig. 3G) and *Bacteroides* (Fig. 3H) were significantly (P < 0.05) lower in all HF groups with respect to the STD group. The supplementation with d-fagomine (the HF + FG and HF + FG + ω-3 groups) partially counteracted the reduction in the relative populations of *Prevotella* (Fig. 3G). The populations of *Bacteriodes* was significantly affected only in the doubly supplemented rats (Fig. 3H).

In cecal samples, the proportion of Bacteroidetes significantly (P < 0.01) increased in the group of animals supplemented with both d-fagomine and ω-3 PUFAs, with respect to all the other groups (Fig. 4A). The proportion of Firmicutes significantly increased in the HF group and those groups given single supplementations with respect to the STD group, while the group supplemented with both d-fagomine and ω-3 PUFAs showed values similar to the STD group (Fig. 4B). The highest Bacteroidetes/Firmicutes ratio resulted from the combined supplementation (the HF + FG + ω-3 group, Fig. 4C). The percentage of Lactobacilliales (Fig. 4D) was significantly (P < 0.01) higher in the HF + FG group than in the HF and HF + ω-3 groups; while the population of Bifidobacteriales (Fig. 4E) in both d-fagomine-supplemented groups was not significantly different from that in the STD group. The proportion *Bacteroides* was significantly (P < 0.05) higher in animals supplemented with both d-fagomine and ω-3 PUFAs than in those given the high-fat diet (Fig. 4H).

Animals in the HF group as well as those given single supplementation (HF + FG and HF + ω-3 groups) had significantly lower cecum content than those in the STD group. The group supplemented with both d-fagomine and EPA/DHA 1:1 had significantly higher (P < 0.01) cecum content than the HF group, and it was statistically similar to that of the STD group (Fig. 4I).

### Short-chain fatty acids

SCFAs were determined in feces (Table 2) and in the cecal content (Table 3) at the end of the study (week 23–24).

**Table 2:** Fecal short-chain fatty acids (SCFAs) after week 23 of the study.

	STD		HF		HF + FG		HF + ω-3		HF + FG + ω-3	
	Mean	SEM	Mean	SEM	Mean	SEM	Mean	SEM	Mean	SEM
Acetic acid	115	13	77**	3	91	11	85*	6	80**	4
Propionic acid	13.4	0.9	5.7***	0.5	8.6*	1.6	4.7***	0.9	4.0***	0.8
Isobutyric acid	2.7	0.2	1.0***	0.2	1.4***	0.2	1.3***	0.3	1.6**	0.7
Butyric acid	17	2	8**	1	7***	2	7***	1	6***	2
Isovaleric acid	1.7	0.2	0.6***	0.1	0.9*	0.2	0.4***	0.3	0.5***	0.3
Valeric acid	1.4	0.1	0.8**	0.0	0.9*	0.1	0.6***	0.3	0.6***	0.3
TOTAL SCFAs	152	9	94**	3	110	14	99*	2	93**	2

Data are presented as means with their standard errors; n = 9 per group. SCFA amounts are given in millimoles per kilogram feces. Comparisons were conducted using one-way ANOVA and Tukey’s multiple comparisons test. *P < 0.05, **P < 0.01, ***P < 0.001 *vs* STD.

**Table 3:** Cecal short-chain fatty acids (SCFAs) at the end of the study (24 weeks).

	STD		HF		HF + FG		HF + ω-3		HF + FG + ω-3	
	Mean	SEM	Mean	SEM	Mean	SEM	Mean	SEM	Mean	SEM
Acetic acid	160	18	75***	9	84***	10	89***	10	74***	8
Propionic acid	32.4	3.7	25.5	2.6	32.4	3.2	32.4	4.2	34.0	5.4
Isobutyric acid	3.7	0.3	2.1*	0.1	3.4	0.3	2.6*	0.2	3.5	0.4
Butyric acid	12	1	25**	2	25**	3	33***	3	27***	3
Isovaleric acid	3.2	0.3	1.9	0.2	3.6^&^	0.4	2.7	0.4	3.2	0.6
Valeric acid	1.9	0.3	3.4	0.2	4.6***	0.4	4.7***	0.4	4.4***	0.4
TOTAL SCFAs	213	23	133*	13	153	14	164	17	147	16

Data are presented as means with their standard errors; n = 9 per group. SCFA amounts are given in millimoles per kilogram cecum. Comparisons were conducted using one-way ANOVA and Tukey’s multiple comparisons test. *P < 0.05, **P < 0.01, ***P < 0.001 *vs* STD; and ^&^P < 0.05 *vs* HF.

In fecal samples, the concentration of total SCFAs and the major component (acetic acid) was significantly reduced (P < 0.05) in all HF groups except the group supplemented only with d-fagomine.

In cecal samples, the concentration of acetic acid was significantly reduced (P < 0.001) in all HF groups; while the concentration of butyric acid was significantly higher (P < 0.01) in all these groups than in the STD group. The levels of isobutyric acid were significantly reduced (P < 0.05) in the HF group without supplementation and the group supplemented only with ω-3, but not in the 2 d-fagomine supplemented groups. The concentration of isovaleric acid was significantly higher (P < 0.05) in the HF + FG group than in the HF group; while levels of valeric acid were higher (P < 0.001) in all the supplemented groups (HF + FG, HF + ω-3 and HF + FG + ω-3) than in the STD group.

### Liver histology

**Figure 5:**
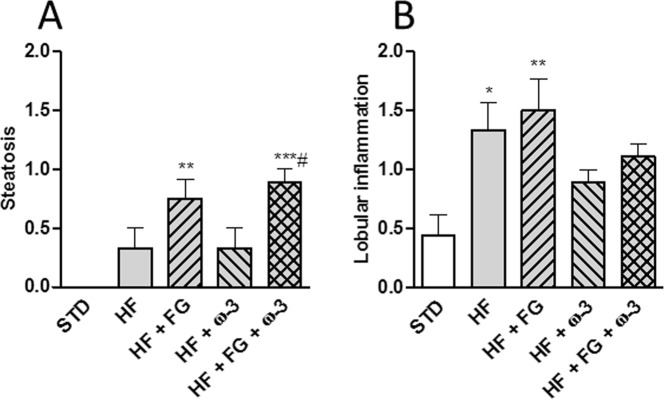
Liver histology. Estimation of variables in SD rats fed a standard (STD), high-fat (HF), high-fat supplemented with d-fagomine (HF + FG), high-fat supplemented with EPA/DHA (1:1) (HF + ω-3) or high-fat supplemented with both d-fagomine and EPA/DHA (1:1) (HF + FG + ω-3) diet at the end of the study (week 24). Steatosis (**A**) and lobular inflammation. (**B**) Scores are presented as means with their standard error. Comparisons were made using one-way ANOVA followed by Tukey’s post-hoc test. *P < 0.05, **P < 0.01, ***P < 0.001 *vs* STD; and ^#^P < 0.05 *vs* HF.

Steatosis and lobular inflammation were determined by histology (Fig. 5). The livers of animals supplemented with d-fagomine presented higher (P < 0.05) steatosis than those from the STD group (Fig. 5A) and the livers from animals supplemented with both d-fagomine and ω-3 PUFAs presented higher (P < 0.05) steatosis than those from rats in the HF and HF + ω-3 groups. Lobular inflammation of animals in the HF and HF + FG groups was higher (P < 0.05) than for those in the STD group (Fig. 5B).

## Discussion

The present study examines the effects of the combination of d-fagomine and ω-3 PUFAs (EPA/DHA 1:1) on SD rats at a very early stage in the development of fat-induced pre-diabetes. SD rats aged 8–9 weeks and fed a high-fat diet (HF group) developed incipient IR as assessed by plasma insulin levels (P < 0.01 vs. STD group) HOMA-IR (P < 0.05 vs. STD group) (Fig. 2). They showed significantly high perigonadal fat accumulation (P < 0.05 vs. the STD group) without reaching statistically significant differences in weight gain (P = 0.07 vs. STD group) over a period of 24 weeks. These changes are consistent with a physiological compensation for reduced insulin sensitivity, the first stage in the progression to diabetes proposed for humans^4^. SD rats did not develop significant IGT (Supplementary Material, Fig. 2). The metabolic effects of the high-fat diet are less evident than those we reported for Wistar Kyoto (WKY) rats^13^. WKY rats fed a high-fat diet significantly (P < 0.001) gained more weight than those fed a STD diet over the same period of time^13^ and they presented IGT with postprandial blood glucose levels around 150 mg/dL, already at week 13 of the intervention^13^; while here, postprandial blood glucose concentration in SD rats fed a high-fat diet was lower than 120 mg/dL even at week 20 (Supplementary Material, Fig. 2). These observations coincide with the results published by other authors showing that: (i) even though SD rats gain more weight than Wistar rats, the differences between the STD group and the high-fat group are lower in SD rats; and (ii) SD rats fed a high-fat diet develop IGT later than Wistar rats do^20^. Therefore, differences in weight gain and effects on glucose metabolism are harder to detect in SD rats than in Wistar or WKY rats.

d-Fagomine showed some effect on IR both when given alone and combined with EPA/DHA 1:1 as the values of HOMA-IR were statistically similar to those in the controls (STD group) (Fig. 2). The groups supplemented with d-fagomine also presented a level of perigonadal fat statistically similar to the STD group (Fig. 1B). The increase of visceral adiposity in the HF group corresponded to the increase in the levels of leptin (Fig. 1C). The anorexigenic hormone leptin is mainly secreted by adipocytes and it is directly related to adipose tissue mass^28^. Plasma leptin concentration in the supplemented groups was statistically similar to those in the STD group (Fig. 1C). In agreement with our previous results^13^, fat accumulation and IR did not increase systemic OS as assessed by the levels of urine F_2t_-IsoPs (Fig. 1D). This result is also consistent with the observation that the generation of mitochondrial ROS occurs later than IR as a consequence of hyperglycemia, as observed in skeletal muscle of mice fed a high-fat high-sucrose diet^29^.

As there is mounting evidence that alterations in gut microbiota (dysbiosis) and intestinal barrier dysfunction may be the first steps leading to dietary fat-induced chronic systemic low-grade inflammation, visceral fat accumulation and IR^30^, we checked the effects of d-fagomine, ω-3 PUFAs and their combination on relevant bacterial subgroups. At the *phyla* level, d-fagomine counteracted the high-fat diet-induced changes in the excreted populations of Bacteroidetes and Firmicutes (Fig. 3A,B). These results confirm our previous observations in WKY rats^13^. When administered alone, ω-3 PUFAs did not affect the changes in the excreted populations of Bacteroidetes and Firmicutes triggered by the high-fat diet (Fig. 3A,B). The combination of both supplements resulted in the highest Bacteroidetes/Firmicutes ratio in the cecum (Fig. 4C).

We next evaluated the changes in the populations of the putatively beneficial Lactobacilliales, Bifidobacteriales and the opportunist Enterobacteriales. d-Fagomine promoted the growth of Lactobacilliales and Bifidobacteriales (Fig. 3D,E, respectively) in agreement with our previous study in rats fed a standard diet^14^. Bifidobacteria have been shown to protect gut barrier function by preserving mucosal permeability and preventing translocation of pro-inflammatory pathogenic enterobacteria, such as *E*. *coli*^31^. The high-fat-induced state of systemic low-grade inflammation is probably initiated by bacterial components such as LPS which leak from an altered intestinal barrier^32^. d-Fagomine might be exerting an anti-inflammatory action by fostering gut colonization with bifidobacteria. d-Fagomine may also contribute a direct action against putatively harmful enterobacteria as it is capable of inhibiting adhesion of *E*. *coli* to the intestinal mucosa^11^. Neither the excess of fat nor the supplementation with ω-3 PUFAs had any significant effect on excreted lactobacilli or bifidobacteria.

It is known that part of the beneficial health effects of a well-balanced gut microbiota is mediated by SCFAs, which are products of microbial fermentation of dietary fiber^33^. The total excreted SCFAs were lower in the HF groups than in the STD group. This may be due to the fact that cellulose is almost the only source of fiber in the high-fat diet, which has been proved to yield amounts of SCFAs as low as those generated by fiber-free diets^33^. High-fiber low-fat diets are known to generate large amounts of fecal SCFAs (mainly acetic acid) compared to fiber-poor diets^34^. d-Fagomine partially counteracted the decrease of acetic acid and total SCFAs in rats fed the high-fat diet (Table 2). This effect could be explained by d-fagomine promoting the growth of SCFA-producing bacteria. We recently reported that d-fagomine stabilizes the populations of the genus *Prevotella* in the intestinal tract of SD rats fed a standard diet while reducing weight gain (Hereu, *Nutrients*, accepted). In the present study, d-fagomine showed some capacity to counteract the high-fat-induced reduction in the populations of *Prevotella* (Fig. 3G). As bacteria from this genus are capable of fermenting complex polysaccharides from the diet in a process that is mechanistically linked to functional effects on glucose metabolism^35^, we hypothesize that d-fagomine might collaborate with dietary fiber in promoting the growth of beneficial bacteria. This effect is necessarily weak in the present case because the high-fat diet is low in fermentable fiber. The weight of the cecum is another indication of gut microbial activity, as rats supplemented with dietary fiber have been reported to have both cecal content and cecal tissue increased already after just two weeks of intervention^36,37^. Here, the cecal content in the HF group was significantly reduced with respect to the STD group while the cecal weight in the group supplemented with both d-fagomine and ω-3 PUFAs was significantly higher than that in the HF group and similar to that in the STD group. The present results with bacterial subpopulations and their fermentation products provide evidence that d-fagomine may contribute to the prevention of metabolic alterations by promoting balanced gut microbiota, whether it is combined with ω-3 PUFAs or not. The combination of supplements appears to be more effective than the individual supplementations in some cases (e.g. the Bacteroidetes:Firmicutes ratio and cecal content).

Liver histology yielded contradictory results, which may be consequence of activities downstream of gut microbial changes. The high-fat diet triggered lobular inflammation as expected^13^. d-Fagomine did not counteract this effect (Fig. 5B). The pro- and anti-inflammatory effects of the different agents in this experimental set-up may provide an explanation for these results. We previously showed that d-fagomine reduced the levels of fat-induced inflammatory markers in plasma and liver and we postulated that these effects might be attained by re-balancing the intestinal microbiota^13^. One possible explanation for the lack of effect of d-fagomine on SD rats fed a high-fat diet reported here is that the animals not supplemented with ω-3 PUFAs (STD, HF and HF + FG groups) were administered an equivalent dose of soybean oil to compensate for both the stress of probing and the excess of calories. Soybean oil, which is rich in the ω-6 PUFA LA, might have counteracted the putative microbiota-related anti-inflammatory effect of d-fagomine. LA may increase the levels of inflammatory prostaglandins and cytokines by entering the ARA metabolic pathway (for a review see^38^). This will not happen in the case of ω-3 PUFA supplementation, as EPA and DHA are converted into less inflammatory and even anti-inflammatory metabolites (for a review on the actions of ω-6 and ω-3 PUFAs see^39^). These events would occur downstream of the fat-induced microbial-triggered stimulation of inflammatory pathways. LA may have contributed to liver inflammation in the HF + FG group while EPA/DHA 1:1 might have reduced the pro-inflammatory effect of the saturated fats in the diet. The picture that emerges from this study, together with our previous reports, points towards a functional role for d-fagomine in the maintenance of eubiosis and the prevention of diet-induced IR reinforced by the action of ω-3 PUFAs via complementary mechanisms.

In conclusion, combined d-fagomine and EPA/DHA 1:1 attenuate high-fat-induced visceral fat, incipient IR, and liver inflammation; while increasing the cecal content, the Bacteroidetes:Firmicutes ratio and the gut population of Bifidobacteriales. The functional effects of the combination appear to be mainly those of d-fagomine complemented by those of EPA/DHA 1:1 (See Table 4 for a summary).

**Table 4:** Qualitative summary of the main effects of diet and supplementations in SD rats compared to rats fed a standard diet.

	Fasting Glucose	Fasting Insulin	HOMA-IR	B:F^£^ ratio	Bifidobacteriales	Lactobacilliales
HF	↑	↑	↑	≈	≈	≈
HF + FG	↑	≈	≈	≈	↑	↑
HF + ω-3	↑	≈	↑	≈	≈	≈
HF + FG + ω-3	≈	≈	≈	↑	↑	≈

^£^Bacteroidetes: Firmicutes.

## Supplementary information

Supplementary Information
