# Supplementary material for: Effects of combined d-fagomine and omega-3 PUFAs on gut microbiota subpopulations and diabetes risk factors in rats fed a high-fat diet
Source: Sci Rep. 2019 Nov 12;9:16628. doi: 10.1038/s41598-019-52678-5 (PMC6851385; doi:10.1038/s41598-019-52678-5)
Supplement: Supplementary file 1 — Supplementary Information [file 41598_2019_52678_MOESM1_ESM.docx]

**SUPPLEMENTARY INFORMATION**

**Effects of combined d-fagomine and omega-3 PUFAs on gut microbiota subpopulations and diabetes risk factors in rats fed a high-fat diet**

Mercè Hereu^1^, Sara Ramos-Romero^1,2*^, Cristina Busquets^1^, Lidia Atienza^3^, Susana Amézqueta^4^, Bernat Miralles-Pérez^5^, Maria Rosa Nogués^5^, Lucía Méndez^6^, Isabel Medina^6^, Josep Lluís Torres^1^

^1^ Institute of Advanced Chemistry of Catalonia (IQAC-CSIC), Barcelona, Spain

^2^ Department of Cell Biology, Physiology & Immunology, Faculty of Biology, University of Barcelona, Barcelona, Spain

^3^ Department of Pathology Puerta del Mar University Hospital, Cádiz, Spain

^4^ Departament d’Enginyeria Química i Química Analítica and Institut de Biomedicina

(IBUB), Universitat de Barcelona, Barcelona, Spain

^5^ Universitat Rovira i Virgili, Unitat de Farmacologia, Facultat de Medicina i Ciències de la Salut, Reus, Spain

^6^ Instituto de Investigaciones Marinas (IIM-CSIC), Vigo, Spain

* **Corresponding Author**

Dr. Sara Ramos-Romero

IQAC-CSIC

Jordi Girona 18-26, 08034 Barcelona, Spain

Phone: (+34) 934006100, Fax: (+34) 932045904,

E-mail: [sara.ramos@iqac.csic.es](mailto:sara.ramos@iqac.csic.es)

Table 1.- Composition of the experimental diets.

|  | **Diets** | | |
| --- | --- | --- | --- |
|  | **Standard^a^** | **High-fat^b^** | **High-fat with**  **d-fagomine** |
| **Composition (g/kg)** |  |  |  |
| Protein | 143.00 | 195.00 | 195.00 |
| L-cystine | 3.00 | 3.00 | 3.00 |
| Carbohydrate available | 480.00 | 476.00 | 476.00 |
| Sucrose | - | 340.00 | 340.00 |
| Corn starch | - | 56.86 | 56.86 |
| Maltodextrin | - | 60.00 | 60.00 |
| Crude fiber | 41.00 | 50.00^c^ | 50.00^c^ |
| Fat | 40.00 | 230.00 | 230.00 |
| Anhidrous milkfat | - | 210.00 | 210.00 |
| Soybean oil | - | 20.00 | 20.00 |
| Mineral | 28.37 | 43.00 | 43.00 |
| Vitamin | 1.20 | 19.00 | 19.00 |
| Ash | 47.00 | 35.00 | 35.00 |
| Choline bitartrate | 1.00 | 3.00 | 3.00 |
| d-fagomine ^d^ | - | - | 0.96 |
|  |  |  |  |
| **Macronutrients^e^** |  |  |  |
| Protein (% by weight) | 14.30 | 17.00 | 17.00 |
| Carbohydrate available (% by weight) | 48.00 | 47.60 | 47.60 |
| Fat (% by weight) | 4.00 | 23.00 | 23.00 |
| Energy from protein (%) | 20.00 | 14.70 | 14.70 |
| Energy from carbohydrate (%) | 67.00 | 40.70 | 40.70 |
| Energy from fat (%) | 13.00 | 44.60 | 44.60 |
| Total energy density (Kcal/g) ^f^ | 2.90 | 4.70 | 4.70 |

^a^ Teklad Global 14% protein rodent maintenance diet (2014) from Envigo

^b^ 45% fat diet (TD 08811) from Envigo

^c^ from cellulose

^d^ d-fagomine (Batch:FG1008E) from Bioglane (Barcelona, Spain).

^e^ Values are calculated from ingredient analysis or manufacturer data.

^f^ Energy density is estimate based on the Atwater factors, assigning 4 Kcal/g to protein, 9 Kcal/g to fat, and 4 Kcal/g to available carbohydrate.

Table 2.- Quantitative real-time PCR primers and conditions

| Target bacteria | | Annealing temperature (ºC) | Sequences (5’-3’) | Positive Control | Reference |
| --- | --- | --- | --- | --- | --- |
| Total Bacteria | | 65 | F: ACT CCT ACG GGA GGC AGC AGT | (a) | ^41^ |
|  | |  | R: ATT ACC GCG GCT GCT GGC |  |  |
| Bacteroidetes | | 62 | F: ACG CTA GCT ACA GGC TTA A | *Bacteroides fragilis* | ^42^ |
|  | |  | R: ACG CTA CTT GGC TGG TTC A |  |  |
| Firmicutes | | 52 | F: CTG ATG GAG CAA CGC CGC GT | *Ruminococcus productus* | ^43^ |
|  | |  | R: ACA CYT AGY ACT CAT CGT TT |  |  |
| Lactobacillales | | 60 | F: AGC AGT AGG GAA TCT TCC A | *Lactobacillus acidophylus* | ^44^ |
|  | |  | R: CAC CGC TAC ACA TGG AG |  |  |
| Bifidobacteriales | | 55 | F: CTC CTG GAA ACG GGT GG | *Bifidobacterium longum* | ^45^ |
|  | |  | R: GGT GTT CTT CCC GAT ATC TAC A |  |  |
| Enterobacteriales | 60 | | F: ATG GCT GTC GTC AGC TCG T | *Escherichia coli M15* | ^41^ |
|  |  | | R: CCT ACT TCT TTT GCA ACC CAC T |  |  |
| *Prevotella* | 60 | | F: CAG CAG CCG CGG TAA TA | *Prevotella copri* | ^25^ |
|  |  | | R: GGC ATC CAT CGT TTA CCG T |  |  |
| *Bacteroides* | 60 | | F: GGT TCT GAG AGG AGG TCC C | *Bacteroides fragilis* | ^25^ |
|  |  | | R: GCT GCC TCC CGT AGG AGT |  |  |

^a^ Positive control of Total Bacteria consisted of the bacteria the result was determined for.

A) B)

Figure 1.- Body weight (A, B) of SD rats fed a standard (STD), high-fat (HF), high-fat supplemented with d-fagomine (HF+FG), high-fat supplemented with EPA/DHA (1:1) (HF+ω-3) or high-fat supplemented with both d-fagomine and EPA/DHA (1:1) (HF+FG+ω-3) diet for 24 weeks. Data are presented as means with their standard errors.

A)

B)

C)

Figure 2.- Glycemic response (OGTT curves) in SD rats fed a standard (STD), high-fat (HF), or high-fat supplemented with d-fagomine (HF+FG) diet (A); STD, HF or high-fat supplemented with EPA/DHA (1:1) (HF+ω-3) diet (B); and STD, HF or high-fat supplemented with d-fagomine and EPA/DHA (1:1) (HF+FG+ω-3) diet (C) after ingestion of a single dose of glucose (1 g/kg body weight) at week 20 of the intervention. Data are presented as means with their standard errors.
